# Altered Anterior Insular Asymmetry in Pre-teen and Adolescent Youth with Autism Spectrum Disorder

**DOI:** 10.18314/abne.v1i1.1120

**Published:** 2018-06-13

**Authors:** Cohen JD, Smith T, Thompson K, Collins A, Knaus TA, Tager-Flusberg H

**Affiliations:** 1 Department of Psychology, Xavier University of Louisiana, New Orleans, LA, USA; 2 Department of Chemistry, Xavier University of Louisiana, New Orleans, LA, USA; 3 Department of Neurology, Louisiana State University Health Sciences Center-New Orleans, LA, USA; 4 Department of Psychological and Brain Sciences, Boston University, Boston, Massachusetts, LA, USA

**Keywords:** Autism, Insular cortex, Asymmetry, Morphometry, Development, Autonomic nervous system

## Abstract

Autism Spectrum Disorder (ASD) is hallmarked by social-emotional reciprocity deficits. Social-emotional responding requires the clear recognition of social cues as well as the internal monitoring of emotional salience. Insular cortex is central to the salience network, and plays a key role in approach-avoidance emotional valuation. Consistent right anterior insular hypoactivity and variable volumetric differences of insular cortical volumes were shown previously. The current study analyzed anterior and posterior insular volume/asymmetry changes in ASD across age. Age was used as an additional grouping variable as previous studies indicated differential regional volume in ASD individuals before and after puberty onset. In the current sample, pre-teen ASD expressed left lateralized anterior insula, while adolescent ASD had right lateralization. Typically developing (TD) individuals expressed the opposite lateralization of anterior insula in both age-groups (right greater than left anterior insular volume among pre-teen TD and left greater than right anterior insular volume among adolescent TD). Social-emotional calibrated severity scores from the ADOS were positively correlated with leftward anterior insular asymmetry and negatively correlated with proportional right anterior insular volumes in ASD. Insular cortex has a lateralized role in autonomic nervous system regulation (parasympathetic control in the left, sympathetic control in the right). Atypical insular asymmetry in ASD may contribute to the development of networks with a diminished salience signal to human faces and voices, and may lead to more learned passive avoidant responses to such stimuli at younger ages, leading to more distressed responses in adolescence. Data here supports the use of early behavioral intervention to increase awareness of and reward for social-emotional cues.

## Introduction

Autism spectrum disorder (ASD) is characterized by impaired social communication, social reciprocity, and repetitive stereotypic behaviors [1]. Deficits in social-emotional reciprocity are wide ranging, from abnormal social approach and failure of normal back-and-forth conversation; to reduced sharing of interests, emotions or affect; and failure to initiate or respond to social interactions [2]. Other nonverbal communicative behaviors used for social interaction are also deficient, including abnormalities in eye contact and body language or deficits in understanding and use of gestures. Many of these behaviors rely on accurate recognition of salient social cues to appropriately guide behavior. One of the core functions of the insula is the detection of novel salient stimuli [3–7], including social [8,9] and affective stimuli [10,11], that are important for guiding higher-order mental processes to direct response behavior [12–14]. The anterior insula is central to a broader salience network, important for integrating visceral, autonomic, and hedonic “markers,” [15] and guiding cognitive resources towards goal-directed behavior, including emotional responses [16–20]. Based upon existing knowledge of insular functions, there appears to be significant overlap between characteristic deficits in ASD and information processed by insular cortex. This makes insular cortex a particular region-of-interest in ASD. The current study explored potential differences in insular volume and left-right asymmetry in well-matched groups of ASD and typically developing pre-teen and adolescent youth, and the relationships of these measures of the insula with the severity of social reciprocity deficits seen in individuals with ASD.

The insula is unique in that it is situated at the interface of the cognitive, homeostatic, and affective systems of the human brain [21]. It, therefore, provides a link between stimulus-driven processing and brain regions involved in monitoring the visceral (anterior insula) as well as the physical state of the internal body (posterior insula) in relation to the outside environment [22,23]. The insula is a functionally heterogeneous brain region, organized by its cytoarchitectonics [24]. The anterior insula significantly contributes to the experience of emotion from information about bodily states via signals from the autonomic nervous system (ANS) [25]. In fact, anterior insula is lateralized in its ANS functionality. Left anterior insula has greater control over the activation of the parasympathetic nervous system, whereas the right anterior insula is involved in the activation of the sympathetic nervous system [26]. The anterior insula has dense reciprocal connections with amygdala and moderate connections with superior temporal sulcus (STS), while the posterior insula has little to no connectivity with these regions [27,28].

Recent evidence suggests a role of insular cortex in ASD [29,30]. A meta-analysis of functional neuroimaging studies of social processing ranging from face processing to theory of mind in ASD revealed consistent hypoactivity of right anterior insula. The hypoactivation of right anterior insula may indicate diminished sympathetic tone in ASD, particularly in response to the faces of other people [29]. Recent data suggests that ASD children exhibit diminished autonomic response (i.e., decreased heart rate reactivity) in response to social tasks [30,31]. Furthermore, earlier studies in ASD show mixed results of increased left or decreased left insula and decreased right insula, however these studies used voxel-based morphometry (VBM) [32,33] and did not analyze differences across age groups. Thus, it is possible that functional asymmetry of anterior insula could be reflected in structural asymmetry (i.e., left > right insular volume).

Considering that anterior insula connects to amygdala, it is also noteworthy that amygdalar volumes are enlarged among pre-adolescent children with ASD, but do not undergo typical age-related volume increase resulting in no amygdalar volume differences between ASD and typically developing (TD) adolescents [34]. The amygdala is important in emotion regulation, and altered anatomy is associated with increased autism symptom severity [35]. STS, also connected to anterior insula, shows reduced grey matter volume [36], reduced cortical thickness [37], and hypoactivation in ASD in both facial gaze tasks and auditory tasks involving human voice stimuli [38,39]. To our knowledge, only one previous study found a relationship between insular cortical thickness and social symptom severity [40]. It is possible that anterior insular volumes may be enlarged in younger ASD individuals (similar to amygdala), but may be the same or smaller between ASD and TD adolescents. By examining differences across age-groups between ASD and TD, the potential exists to explain previous disparate insular volumetric results.

The current study directly assessed insular anatomical differences in young (i.e., preteen) and older (i.e., adolescents) individuals with ASD compared to age-matched TD controls via manual morphometry. This approach is ideally suited to capturing inter-individual variability in native space in contrast to VBM, which requires warping images into standard space. It extends earlier work by examining differences across age-groups. The current approach is also different from previous VBM studies by quantifying functional anterior and posterior insular sub-regions, corresponding to functional subdivisions. We hypothesized that anterior insular volumes would be increased in ASD compared to TD in the younger group but show no difference, or be smaller, in adolescence, paralleling age-related amygdalar differences. No posterior insular volume differences were expected. Based on the discrepancy between left and right insular volume changes in previous studies [32,33], we hypothesized that anterior, but not posterior, insular asymmetry (i.e., the proportional difference between left and right ROI volumes; see Methods for details) would be different in ASD compared to TD, potentially including differences across age groups. Given the findings of diminished activation of right insula; it was expected that there would be left greater than right insular asymmetry in ASD, and that left greater than right asymmetry of anterior, but not posterior, insula would correlate with higher social reciprocity symptom scores on the Autism Diagnostic Observation Schedule (ADOS).

## Materials and Methods

### Participants

Participants were recruited as part of a larger study [41] by word of mouth, flyers posted in the community and online, and from other research projects within Boston University School of Medicine. The current sample consisted of 25 pre-teen and adolescent youth with ASD, ages 7–19 years (mean=12.05, SD=3.29) and 17 TD preteen and adolescents, ages 7–19 years (mean=11.85, SD=2.82). Participants in each group were divided into a younger group (pre-teen, ASD n=14, TD n=9), ages 7–11 years and an older group (adolescents, ASD n=11, TD n=8), ages 12–19 years. Twelve years was used as the division between the older and younger groups as this roughly corresponds to the division between pre-puberty and puberty and is similar to cutoffs to classify pre-teen and adolescents used in previous studies [34,41–46]. All participants spoke English as their first language. There were 4 females in the ASD group (3 younger and 1 older) and 3 in TD (2 younger and 1 older).

The Kaufman Brief Intelligence Test (KBIT-II) [47] was administered to assess IQ, except for one younger participant with ASD who was administered the Differential Ability Scales (DAS) [48].

ASD participants were diagnosed based on DSM-IV criteria for autism or PDD-NOS [49] using the Autism Diagnostic Interview-Revised (ADI-R) [50] and the Autism Diagnostic Observation Schedule, Module 3 or 4 (ADOS) [51]. In addition, an expert clinician confirmed that all individuals met criteria for ASD. Individuals with frank neurological damage (determined by a neurologist reading of the brain scan), with a known genetic disorder (e.g., fragile X syndrome), who were born prematurely (less than 35 weeks), or who had experienced seizures within the last three years were excluded from the study. TD individuals also had no history or current diagnosis of developmental, learning, psychiatric, or neurologic disorders.

Participants 18 years and older were informed of the procedures and gave written consent prior to participation in the study. For participants under 18 years old, parents and participants were informed of the procedures and parents gave written consent prior to the child’s participation in the study. Children 12 years and older also provided written assent prior to participation. All data reported here were collected in compliance with the Boston University School of Medicine Institutional Review Board. Subject groups were well matched on sex, age, verbal and non-verbal IQ (Table 1).

### MRI Acquisition and Pre-processing

All participants were trained in a mock scanner prior to the actual MR scanning. Volumetric MR images were acquired on a Philips 3 Tesla Intera scanner. T1-weighted images were obtained as a series of 160, 1 mm gapless sagittal images. Turbo field echo (TFE) was used, with technical factors of: TR=9.9 seconds, TE=4.6, 256×256 pixel matrix, 24 cm field of view, and 8 degree flip angle. Data sets were rotated into alignment in the sagittal, axial, and coronal planes in order to eliminate any head rotation and MRI scans were maintained in real space. Each MRI scan series was assigned a blind number to assure subject confidentiality and to ensure that all volume measurements were performed blind to group and subject.

### MRI measurements

#### Total brain volume

Total brain volume (TBV) was measured with the MEASURE program [52]. The Brain Extraction Tool (BET) in FSL [53] was first used to remove as much skull as possible, without removing any brain. The contrast was then set and the MEASURE program automatically outlined the brain. The outlining was then manually edited in each slice to remove any remaining non-brain regions from the outline. This measurement included gray and white matter and the thalamus, but excluded the cerebellum and brainstem. For inter-rater reliability, a subset of 5 brains (10 hemispheres) was measured by 4 investigators. An intra-class correlation (ICC) was calculated between the investigators’ volume measurements across both hemispheres of the 5 brains. The ICC for total brain volume was .97.

#### Insular cortex

The insula was manually delineated using the sagittal and coronal planes for each participant according to previously described methods [54], using grayscale volume stacks derived from whole-brain analysis. Insular regions-of-interest (ROI) were traced on the non-segmented grayscale images using BrainImageJava (BIJ) [55] (CIBSR.stanford.edu/tools). The reliability of the insular ROIs was established by achieving an ICC greater than 0.93. (For full insular protocol see [54])

The anterior and posterior boundaries of the insula were viewed best from the sagittal plane. The coronal plane was utilized to define the superior, inferior and medial boundaries of the superior sulcus, inferior sulcus, and extreme capsule respectively.

In addition to total insular volume measurement, an important component of this method is segmentation of the insula into connectivity-based (i.e., functionally relevant) anterior and posterior sub-regions. BIJ has a built in algorithm that creates anteroventral (anterior) and posterodorsal (posterior) ROIs from the total insular ROI [54]. All three (total, anterior, posterior) ROIs were used for analyses.

### Statistical analyses

#### Insular volume

To control for total brain size, total brain volume was used as covariates in all analyses of insular volume. A multivariate analysis of covariance (MANCOVA) was used to examine insular volume differences with diagnosis (ASD, TD) and age group (pre-teen, adolescent) as between subject variables, and left and right anterior, and posterior insular volumes as the dependent variables.

#### Asymmetry Quotients

Left-right asymmetry was quantified as an asymmetry quotient. Asymmetry quotients (AQ) (Left-Right/[(Left+Right)/2]) were calculated for each insular ROI (anterior and posterior). Thus, a positive AQ value indicated left lateralization and negative AQ indicated right lateralization. Insular AQs were analyzed in separate ANOVA analyses with diagnostic group and age group as the between subject variables and the insular ROIs as the dependent variables.

### Behavioral Symptoms and Anatomical Correlations

Pearson correlations were computed to examine the relationship between behavioral and anatomical measures. For all correlations, anterior/posterior insular regional volumes proportional to total hemisphere volume were used (e.g., right proportional anterior insular volume=[raw right anterior volume]/[total right hemisphere volume]). AQs of anterior (primary correlation of interest) and posterior (tested to verify no relationship) insular regions were also correlated with behavioral symptoms. To determine if left or right volumes disproportionately contributed to AQ results, correlations were computed between left and right volumes of anterior and posterior regions with autism symptom severity in the ASD groups. Symptom severity was measured using the communication and social composite severity algorithm scores (CSS) from the ADOS Module 3 [56] or 4 [57]. Higher ADOS CSS reflect increased symptom severity. Correlations were not corrected for multiple comparisons.

## Results

### Insular volumes

All statistical analyses were conducted in SPSS 19. Mean volumetric insular data are given in table 2. The MANCOVA revealed no significant main effects of diagnosis or age-group, and there were no significant interactions for anterior or posterior insular volumes (at the multivariate level, all *p*’s > 0.151). Anterior and posterior insular volumes were not statistically different between ASD and TD individuals of any age or in either hemisphere (left, right).

### Insular asymmetry quotients

**Figure 1:**
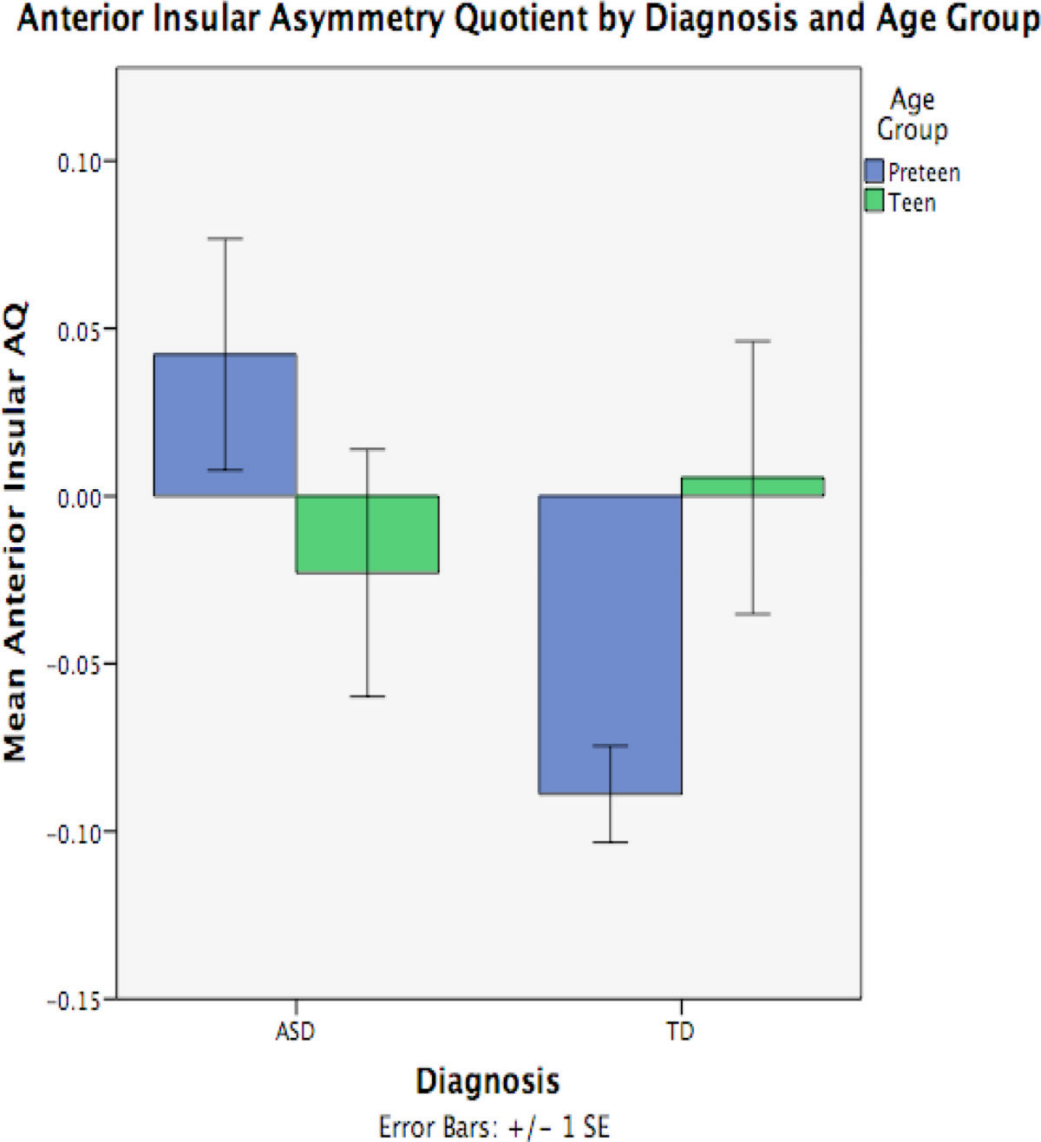
Anterior Insular Asymmetry Quotient Group by Age Group Interaction. Mean AQs for anterior insular cortex by diagnostic group and age group (ASD Pre-teen (N=14), Adolescent (N=11); TD Pre-teen (N=9), Adolescent (N=8)). Positive AQs denote leftward asymmetry and negative AQs denote rightward asymmetry. There was a significant diagnosis-by-age group interaction for anterior insular AQ (F1,37=5.121, p=.029, η2=.12).

Mean AQ’s for each insular region are listed in table 2 (analyses focused on anterior and posterior insula specifically). There were no main effects of diagnosis or age-group in AQ for either insular ROI (all *p*’s > 0.153). There was a significant diagnosis-by-age group interaction for anterior insular AQ (F_1,37_=5.121, *p*=.029, η^2^=.12) (Figure 1). Specifically, the difference in AQ among ASD and TD pre-teens was statistically significant (F_1,21_=8.542, *p*=.008), whereas the AQ difference among adolescents was not (F_1,17_=0.262, *p*=.615). Pre-teen ASD youth exhibited left lateralization of anterior insula compared to a right lateralization among TD pre-teens. While not statistically different, adolescents had the reverse lateralization; right lateralized among ASD and left lateralized among TD. No significant results were found with respect to posterior insular AQ.

### Behavioral symptom correlations

**Figure 2:**
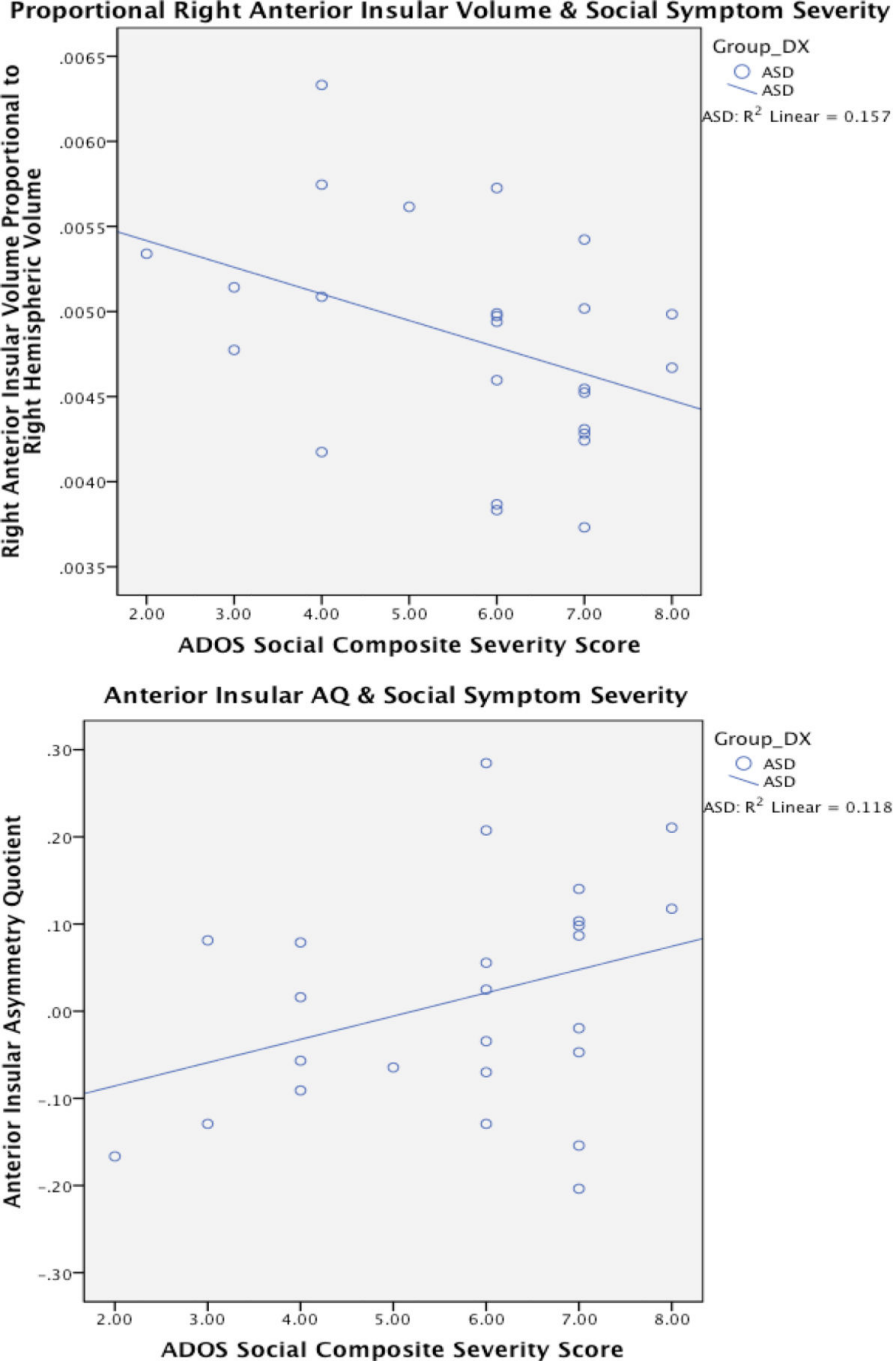
ADOS Social Symptom Severity Correlation with Anterior Insula. Scatter plots of right anterior insular volume (p=.025), proportional to right hemispheric volume, and anterior insular AQ (p=.046) plotted against ADOS Social scores in ASD individuals (N=25). Positive AQs denote leftward asymmetry and negative AQs denote rightward asymmetry.

Correlations between ADOS scores and insular anatomy were computed for the ASD group as a whole. Since the anterior insula was the primary ROI, this region was the main focus for correlations with ASD symptom severity (Figure 2). There was a significant positive correlation between anterior insular AQ and the social CSS of ADOS (*r* (23) =0.343, *p*=.046, *r*^*2*^=.118). Social symptom severity (i.e., reduced social reciprocity) increased with greater leftward anterior insular asymmetry.

Correlations between social symptom severity and proportional left and right anterior insular volumes were explored to determine if changes in a particular hemisphere contributed more to the AQ relationship found. This revealed a significant negative correlation between the social CSS of ADOS and right anterior insular volume proportional to right hemisphere volume (*r* (23) =−0.396, *p*=.025, *r*^*2*^=.157). In general, as proportional right anterior insular volume decreased, social symptom severity (i.e., reduced social reciprocity) increased. There was no relationship between proportional left anterior insular volume and social CSS symptom severity. This suggests that greater right insular volume reduction, and not left insular volume enlargement, is principally responsible for the relationship between increased leftward anterior insular AQ and increased social CSS symptom severity. As expected, there was no relationship between posterior insular AQ, or left or right proportional volumes, and social CSS symptom severity.

## Discussion

The objective of the present study was to utilize an established morphometric method for targeted analysis of insular cortex in native space across age in ASD, which has not previously been carried out. There were key neuroanatomical differences as well as significant relationships between anatomical changes and social impairment. While there were no significant left or right anterior and posterior insular volume differences when considering age-related difference across ASD and TD, there was a significant difference of anterior insular AQ between ASD and TD that also varied across age group. Pre-teen ASD youth had the opposite asymmetry compared to TD (left>right vs. right>left, respectively), and this asymmetry pattern was reversed in adolescence (right>left vs. left>right, respectively). There was also a significant correlation between social reciprocity CSS symptom severity from ADOS, and increased leftward AQ of anterior insula. As ADOS social CSS symptom scores increased, anterior insular AQ also increased, indicating the greater the leftward AQ of anterior insula (i.e., the more left volume was greater than right volume), the higher the social CSS symptom scores. As a complimentary finding, there was a negative correlation between right anterior insular volumes (proportional to overall right hemisphere volume) and social CSS symptom scores. This indicated that the more the right anterior insular volume decreased, the more ASD individuals had difficulties with social reciprocity. There is also the added implication that perhaps the leftward shift in anterior insula AQ (and its relationship to social symptom severity) is driven more by right anterior insular volume reduction than left anterior insular enlargement. As expected, no statistically significant results were found regarding posterior insula.

The primary neuroanatomical result was a difference of anterior insular asymmetry between ASD and TD that also varied by age group. This asymmetry difference was a moderately strong effect. The pre-teen children with ASD had a strong leftward asymmetry of anterior insular volume compared to a rightward asymmetry among TD pre-teen youth.

This asymmetry relationship was reversed among adolescent individuals as ASD teenagers expressed a rightward asymmetry of anterior insular volumes compared to leftward asymmetry among TD teenagers. Compared to TD, the shift in asymmetry from pre-teen to teenagers among ASD was influenced by enlarged left anterior insular and reduced right anterior insular volumes in the younger group, and smaller left combined with similar right anterior insular volumes in the older group. The enlarged left volumes in pre-teens along with reduced left volumes amongst adolescents helps elucidate mixed left insular volume differences of previous VBM data, which did not explicitly analyze differences across age [32,33]. Functionally, insular cortex is a key region of the central autonomic network with lateralized control of sympathetic (right) and parasympathetic (left) autonomic tone [26,21]. Individuals with ASD have atypical leftward asymmetry in anterior insula, which may contribute to decreased behavioral reactivity; and is mirrored by both diminished autonomic reactivity [31] and right insular hypoactivity [29,30], in response to socially relevant stimuli. Anatomical and functional insular changes may be related to broader network connectivity issues that prevent typical social stimuli processing. In turn, socially relevant information is not appropriately re-represented in dorsal anterior insula, which is important for signaling the need for behavioral response by guiding switching away from the self-oriented default mode network (DMN) to the action-oriented central executive network (CEN) [58–61]. The lateralization shift in adolescence supports a greater sympathetically driven ANS in ASD and a greater parasympathetically driven ANS in TD. There is evidence that adults with ASD maintain atypical lateralized insular functional connectivity compared to their TD counterparts [62]. The ANS plays a significant role in emotional processing as well as social responding, suggesting that the shift in anterior insula contributes significantly to behavioral symptoms seen in ASD, including higher rates of anxiety in adolescence in response to a variety of stimuli, including reduced eye-gaze and social interaction [35,63,64].

There were also several significant relationships between insular anatomy and ASD symptom severity measured by ADOS social CSS. There was a significant positive correlation between anterior insular AQ (positive AQ values denote leftward asymmetry) and social symptom severity. That is, the more left lateralized anterior insular volumes were, the more severe ASD symptoms were in the reciprocal social interaction domain. These data may help validate consistent right insular volume reductions in previous VBM data [32,33]. Atypical anterior insular asymmetry, which mirrors amygdalar asymmetry [34], contributes significantly to diminished affective reactivity (i.e., higher ADOS scores on social reciprocity scale) in ASD. Reduced right and enlarged left anterior insular cortices result in younger ASD individuals expressing a diminished response, resulting in a failure to engage active behavioral strategies through the CEN. Functionally, this finding is consistent with recent data showing reduced autonomic responsiveness towards social stimuli in ASD [31].

Using proportional insular volumes in relation to the respective hemisphere volume, there was a significant negative relationship between social symptom severity and right anterior insula. As proportional volume of right anterior insular cortex decreased, ADOS social symptom severity increased. This relationship suggests that reduced right anterior insular volume, as opposed to left anterior insular enlargement may be responsible for the increased leftward anterior insular AQ relationship to greater social reciprocity symptom severity in ASD. Thus, as proportional right anterior insular volumes decreased, more of these social behaviors such as eye contact and facial expressions are lacking. Anterior insular cortex was previously implicated as part of a network sub-serving atypical eye-gaze patterns as well as heightened anxious response to direct eye-gaze [65–67] in Fragile X Syndrome. It is possible the anterior insula is part of a similar network in ASD that may contribute to general deficits in socially reciprocal behaviors. Moreover, the reduction of right anterior insula with increasing social symptom severity could reflect a neuroanatomical correlate of functional hypoactivation of this region during social processing tasks [29].

One interpretation of our data would be that atypical insular asymmetry in ASD may contribute to the development of networks with a diminished salience signal to human faces and voices. This atypical functional network may lead to more learned passive avoidant responses to such stimuli. Conversely, their preteen TD counterparts exhibit a more rightward insular AQ resulting in more mobilized social responses necessary for the development of an intact social engagement system [68] into adolescence (highlighted by greater leftward insular AQ in TD teenagers). Without the benefit of early-learned actively engaged responses to social stimuli, ASD adolescents experience much greater difficulty navigating social interactions once more reactive resources do develop. Emerging data on the benefits of behavioral intervention support this model. By engaging parent-implemented intervention strategies targeting social reciprocity, ASD symptomatic children (around age 1) saw significantly reduced social symptom severity at two, three [69], and six [70] years of age. While individuals with ASD react less to social stimuli [2,31], this low reactivity may be reversed in some cases as a result of early intervention. The data here suggests that anterior insular cortex may play a central role in emotional reactivity capacity to social stimuli in ASD, and may be involved in the success of behavioral intervention by targeting and increasing insular network activation. Atypical anterior insular asymmetry may indeed underlie social reciprocity deficits in ASD, and behavioral intervention may function through the reorganization and functional balance of insular-centered social neural networks.

There are several limitations to the current study. Our TD sample was smaller than the ASD sample, and there were also small numbers within the pre-teen and adolescent ASD and TD subgroups. Ideally, the effects reported here should be repeated in new samples with larger sample sizes. We note, however, all the significant statistical results of this study had moderately strong to strong effect sizes. The age subgroups were also cross sectional as opposed to longitudinal, which is not ideal for elucidating developmental changes. Our sample did not have the power to explore gender effects; therefore, gender effects were not explicitly investigated. Finally, while insular cortex is noted to be involved in anxiety, measures of anxiety were not available to investigate this directly.

Future studies should examine the presence and severity of anxiety symptoms in ASD in relation to insular anatomy and function. In order to increase N, future studies should also aim to utilize machine-learning algorithms in order to translate the reliability of manual tracing methods into the time efficiency of automated imaging to ease the collection of larger insular data sets. As noted above, behavioral intervention reduces autism social symptom severity, potentially by actively engaging mobilization strategies via increasing activation of right anterior insula in particular. Future studies should investigate longitudinal brain changes in ASD, with specific emphasis on changes resulting from behavioral intervention. Emerging evidence also suggests that the use of real-time neurofeedback (via fMRI) targeting anterior insula has benefits in emotional processing, and efforts should be made to apply this approach in ASD [30]. Network-based changes could also be explored by including analysis of amygdalar, insular and other salience network measures in the same study.

## Figures and Tables

**Table 1:** Subject demographic and IQ data.

Group	ASD		TD		Comparisons					
Age-Group	Pre-teen (N=14)	Adolescent (N=11)	Pre-teen (N=9)	Adolescent (N=8)	Diagnosis		Age-group		Interaction	
					F1,37	p	F1,37	p	F1,37	p
TBV (cm3)	1282.34 (142.04)	1338.05 (138.07)	1315.56 (103.71)	1375.98 (190.42)	0.609	0.44	1.622	0.211	0.003	0.959
Age	9.74 (1.58)	14.98 (2.41)	9.68 (1.57)	14.29 (1.59)	0.413	0.524	N/A	N/A	0.291	0.593
Verbal IQ	89.00 (32.87)	111.00 (28.55)	118.44 (10.26)	111.13 (21.13)	3.751	0.06	0.925	0.342	3.688	0.06
Non-Verbal IQ	99.21 (30.87)	105.36 (9.28)	110.56 (7.38)	105.25 (10.47)	0.832	0.367	0.005	0.946	0.866	0.358
Total IQ	94.57 (31.73)	109.73 (15.02)	117.00 (8.99)	109.63 (15.66)	2.684	0.11	0.326	0.571	2.734	0.106
ADOS Communication Composite Severity Score	2.14 (0.949)	2.09 (0.701)	N/A	N/A						
ADOS Social Composite Severity Score	5.79 (1.48)	5.64 (1.91)	N/A	N/A						

Means and standard deviations of group demographic data for age, total brain volume (TBV), verbal, non-verbal, and total IQ scores. ADOS communication and social scores are presented only for ASD individuals, as TD subjects have zero score values. All scores are split across pre-teen and adolescent age groups with ASD and TD diagnostic groups. Comparison columns represent the F-statistics and associated p-values for main effects (Diagnosis, Age-group differences) and Interaction (Diagnosis*Age-group) for ANOVAs testing differences in TBV, verbal, non-verbal, and total IQ scores. The main effect of Age-group was left out for the age difference analysis since the creation of younger and older age groups inherently created groups with statistically difference ages. IQ score differences were tested by Multivariate Analysis of Variance (MANOVA), with each set of scores as dependent variables (DV). All MANOVA statistics were non-significant. Scores listed in the table represent individual statistics for each DV at the univariate level.

**Table 2.** Insular volumetric data and asymmetry quotients.

Group	ASD	TD		
Age-Group	Pre-teen (N=14)	Ado lescent (N=11)	Pre-teen (N=9)	Adolescent (N=8)
Left Anterior Insula	3.14 (0.46)	3.25 (0.40)	3.04 (0.33)	3.33 (0.44)
Right Anterior Insula	3.01 (0.41)	3.34 (0.52)	3.32 (0.31)	3.32 (0.45)
Left Posterior Insula	2.56 (0.33)	2.64 (0.53)	2.39 (0.26)	2.60 (0.30)
Right Posterior Insula	2.48 (0.46)	2.52 (0.36)	2.41 (0.53)	2.76 (0.48)
Left Total Insula	5.71 (0.76)	5.89 (0.87)	5.43 (0.54)	5.93 (0.66)
Right Total Insula	5.49 (0.75)	5.86 (0.75)	5.74 (0.78)	6.08 (0.89)
Anterior AQ	0.042 (0.129)	−0.023 (0.122)	−0.089 (0.043)	0.006 (0.115)
Posterior AQ	0.041 (0.154)	0.037 (0.124)	0.012 (0.261)	−0.051 (0.141)
Total AQ	0.040 (0.100)	0.003 (0.078)	−0.050 (0.102)	−0.019 (0.098)

Mean and standard deviations of uncorrected anterior, posterior, and total insular volumes (in cm3), as well as asymmetry quotients (AQs) for each insular ROI, arranged by diagnostic group and age group. Positive AQs denote leftward asymmetry and negative AQs denote rightward asymmetry.

## Abbreviations

ADOS: Autistic Diagnostic Observation Schedule
ADI-R: Autism Diagnostic Interview-Revised
ASD: Autism Spectrum Disorder
ANOVA: Analysis of Variance
ANS: Autonomic Nervous System
AQ: Asymmetry Quotient
BET: Brain Extraction Tool
BIJ: BrainImageJava
CSS: Composite Severity algorithm Scores
DAS: Differential Ability Scales
ICC: Intra-class Correlation Coefficient
KBIT-II: Kaufman Brief Intelligence Test
MANCOVA: Multivariate Analysis of Covariance
ROI: Region-of-Interest
STS: Superior Temporal Sulcus
TBV: Total Brain Volume
TD: Typically Developing
TFE: Turbo field echo
VBM: Voxel-based Morphometry
